# Effect of Drinking Water Distribution System Design on Antimicrobial Delivery to Pigs

**DOI:** 10.3390/ani11082362

**Published:** 2021-08-10

**Authors:** Stephen Little, Andrew Woodward, Glenn Browning, Helen Billman-Jacobe

**Affiliations:** 1 Asia Pacific Centre for Animal Health, Melbourne Veterinary School, Faculty of Veterinary and Agricultural Sciences, and National Centre for Antimicrobial Stewardship, University of Melbourne, Parkville, VIC 3010, Australia; glenfb@unimelb.edu.au (G.B.); hbj@unimelb.edu.au (H.B.-J.); 2 Melbourne Veterinary School, Faculty of Veterinary and Agricultural Sciences, University of Melbourne, Parkville, VIC 3010, Australia; andrew.woodward@unimelb.edu.au

**Keywords:** drinking water, antibiotic, antimicrobial, medication, water distribution system, hydraulic modelling, pig water usage, dosing pump, dosing regimen, metaphylaxis

## Abstract

**Simple Summary:**

The piped water system in buildings that house growing pigs is used on many farms for short periods to medicate pigs with antimicrobials, in order to keep them healthy and productive. However, the effect that the design of a building’s water system has on antimicrobial delivery to pigs in pens throughout the building is not known. Thus, we tracked the antimicrobial concentration in water available to pigs at four drinkers during four in-water dosing events, each conducted with looped water systems differing in their design. We found that the water system’s design and the pigs’ water usage and drinking patterns had a large influence on water flow and, therefore, the amount of antimicrobial delivered to pigs in each pen over time. We discovered that by using a circulator pump in a building’s looped water system, all pigs within a building could be delivered the same antimicrobial concentration in water over time. We also showed how a hydraulic modelling tool can be used to predict the antimicrobial concentration at drinkers over time in a specific building during a dosing event. This provides an opportunity to compare alternative in-water dosing schedules for pigs in a given building and select the one likely to be the most effective.

**Abstract:**

On many pig farms, growing pigs are mass-medicated for short periods with antimicrobial drugs through their drinking water for metaphylaxis and to treat clinical disease. We conducted a series of four prospective observational cohort studies of routine metaphylactic in-water antibiotic dosing events on a commercial pig farm, to assess the concentration of antimicrobial available to pigs throughout a building over time. Each dosing event was conducted by the farm manager with a differently designed looped water distribution system (WDS). We found that the antimicrobial concentration in water delivered to pigs at drinkers in each pen by a building’s WDS over time was profoundly influenced by the design of the WDS and the pigs’ water usage and drinking pattern, and that differences in the antimicrobial concentration in water over time at drinkers throughout a building could be eliminated through use of a circulator pump in a looped WDS. We also used a hydraulic WDS modelling tool to predict the antimicrobial concentration at drinkers over time during and after a dosing event. Our approach could be used to evaluate alternative in-water dosing regimens for pigs in a specific building in terms of their clinical efficacy and ability to suppress the emergence of antimicrobial resistance, and to determine the optimal regimen. The approach is applicable to all additives administered through drinking water for which the degree of efficacy is dependent on the dose administered.

## 1. Introduction

**Figure 1:**
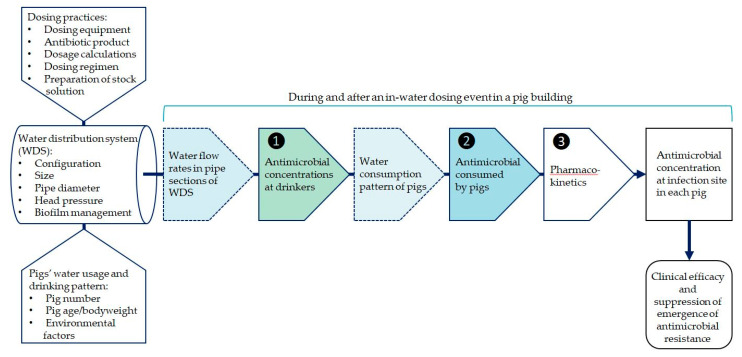
The process of in-water dosing in a pig building, showing three sources of between-animal variability in systemic exposure to an antimicrobial (numbered 1, 2 and 3).

The drinking water distribution system (WDS) in buildings that house growing pigs is used on many farms for short periods to mass-medicate growing pigs with antimicrobials, which are administered into the main water line through a proportional dosing pump or header tank. In-water dosing may be performed strategically at pre-planned intervals for metaphylaxis, and whenever necessary to treat clinical disease caused by bacterial pathogens [1,2,3,4]. Successful in-water dosing of a group of growing pigs in a building should result in the majority of pigs (e.g., 90%) attaining the level of systemic exposure to the antimicrobial required to achieve high clinical efficacy, while minimizing selection for and propagation of resistant pathogens [5,6]. For some classes of antimicrobials (e.g., β-lactams) efficacy is time-dependent, while for others (e.g., aminoglycosides) efficacy is concentration-dependent. For some classes (e.g., tetracyclines) efficacy is dependent on both time and concentration [1]. Several studies have found that, when an antimicrobial is administered to a group of pigs in drinking water offered *ad libitum*, there is substantial variability in the systemic exposure of pigs to the antimicrobial [7,8,9,10,11]. There are three sequential sources of this between-animal variability in the in-water dosing process: differences in (1) the antimicrobial concentration in water delivered to pigs by the WDS; (2) the antimicrobial dose consumed by pigs; and (3) the pharmacokinetics of the antimicrobial, such as its oral bioavailability and plasma clearance (Figure 1) [1]. This study focused on the first of these sources of variability: differences in the antimicrobial concentration in water delivered to pigs at drinkers in each pen over time by the WDS.

When applying in-water dosing, the time course of antimicrobial concentration in water available to pigs at drinkers in each pen is a consequence of the water age (residence time) at each drinker over time. The residence time is a consequence of the water flow rates in each pipe section of the building’s WDS. Water ages and flow rates depend, firstly, on characteristics of the WDS, specifically: its configuration—either looped (in which water travels in two directions) or branched (in which water travels in one direction); the lengths, diameters and internal surface smoothness of the pipes; the number of bends and constrictions or expansions in the diameter of pipes along the WDS due to the fittings that have been installed; the presence of non-return valves, pressure gauges and pressure regulators; and the system’s head pressure [12]. If biofilms and sediments are allowed to accumulate in pipe sections of the WDS, they may also exert hydraulic effects [13]. Water flow rates in each pipe section of the building’s WDS over a 24 h period are also a function of the pigs’ water demand, which is a function of the number of pigs housed in the building, their bodyweight and their drinking patterns. Data on pigs’ water usage (i.e., water consumed and wasted by pigs) and drinking patterns are sparse, but the estimated mean daily water usage is between 60 and 117 mL/kg bodyweight [1,14]. Most drinking events occur during daylight hours and are associated with feeding events. Water usage is characterised by one or two distinct peaks, with several studies observing two peaks—one after sunrise and another in the mid-late afternoon [15,16]. Pigs’ water usage and drinking patterns may be influenced by stress, boredom, hunger, disease, feed type, drinker water flow rate and environmental conditions [15,16,17,18].

Our study was designed to determine how the concentration of antimicrobial in the water available to pigs at drinkers in pens, along the building’s WDS and at varying distances from the dosing pump, changes over time. We conducted a series of four prospective observational cohort studies of routine metaphylactic in-water antimicrobial dosing events in two large, conventional grower/finisher buildings on a commercial pig farm with solid/slatted floored pens. Buildings with looped WDSs were selected for the studies as they are more common than branched WDSs in such buildings. An externally validated WDS modelling tool was used to understand the hydraulic behaviour of the WDS during each dosing event. These tools enable the spatial and temporal variations of water flow, velocity, pressure, water age, source contribution and disinfectant concentration, as well as other hydraulic and water quality parameters throughout WDSs, to be calculated [19,20,21].

## 2. Materials and Methods

### 2.1. Prospective Observational Cohort Studies of In-Water Dosing Events

**Figure 2:**
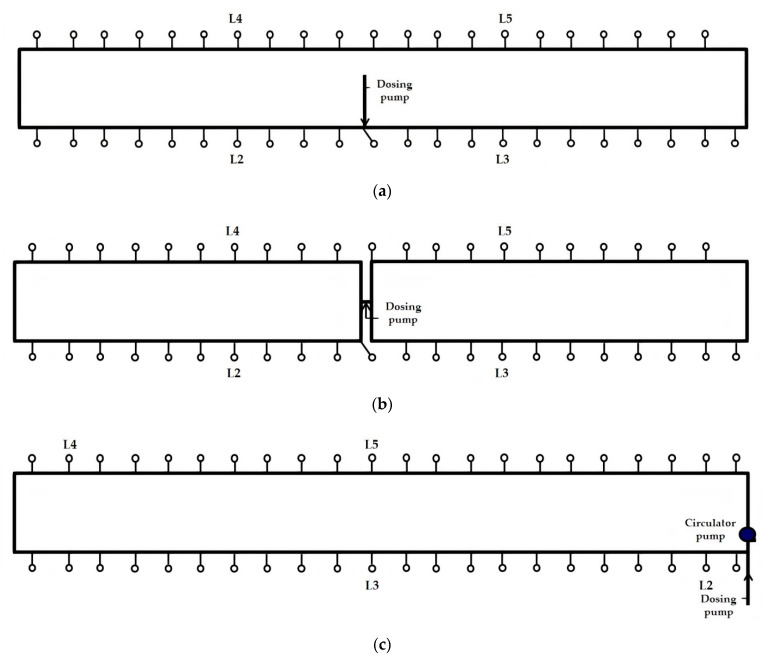
Schematics of looped WDSs in pig grower/finisher buildings: (**a**) Study 1a in Building A, (**b**) Study 1b in Building A, and (**c**) Studies 2a and 2b in Building B. Drinkers are represented with open circles. Drinkers from which water samples were collected for analysis are labelled L2, L3, L4 and L5.

Four studies of routine, metaphylactic in-water dosing events were conducted in two large grower/finisher buildings (Studies 1a and 1b in Building A, and Studies 2a and 2b in Building B). The studies were entirely observational. Each dosing event was conducted by the farm manager using existing farm dosing equipment and practices. Studies 1a and 1b were conducted on consecutive days, as were Studies 2a and 2b. Buildings A and B were the same size and shape, with capacity for up to 2200 pigs. The design of the building’s WDS differed for each of the four studies of dosing events. Building A’s single-looped WDS, as used in Study 1a, had small lengths of pipe and gate valves installed in it prior to the studies which enabled its configuration to be readily changed into two smaller rectangular loops, as used in Study 1b (Figure 2a,b). Building B’s WDS, as used in Studies 2a and 2b, was also a single loop. However, the point at which the water line entered the building, where the dosing pump was positioned, differed from that in Building A (Figure 2c). For Study 2b, a small wet rotor electric circulator pump, pre-installed in the WDS, was run to override the two-directional water flow around the loop and establish a higher velocity, unidirectional (anticlockwise) water flow.

**Figure 3:**
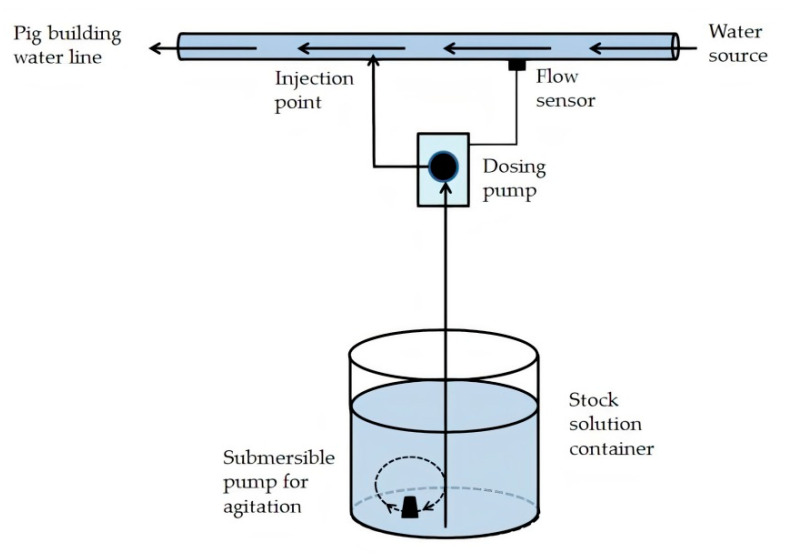
Dosing equipment used in Studies 1a, 1b, 2a and 2b.

In the four studies, pigs were dosed by the farm manager with a soluble antimicrobial product containing amoxicillin (870 g/kg active) as the trihydrate (AbbeyMox Amoxicillin Soluble Powder, Abbey Animal Health Pty. Ltd., Glendenning, NSW, Australia). Amoxicillin is the antimicrobial most commonly administered in-water to growing pigs in Australia [18]. Amoxicillin was injected into the water line using a proprietary electric-powered dosing pump according to the consulting veterinarian’s prescription, applying dosing practices routinely used on the farm. Specifically, a stock solution was prepared in a 70 L plastic container and the dosing pump set to inject at a ratio of 1:100 (*v*/*v*). The stock solution was continually agitated with a small submersible pump (Figure 3).

During each dosing event, a series of 10 mL water samples was collected from each of four drinkers (labelled L2, L3, L4 and L5) located at different distances along each building’s looped WDS from the dosing pump (Figure 2). Samples were collected with the following frequency: 5 min before the dosing event commenced, every 10 min in the first hour, every 15–20 min in the second and third hours, every 30 min until the dosing event finished (when the dosing pump stock solution was fully depleted) and then every 15–20 min for a further 1 to 1.5 h if still within the farm’s working hours. In Studies 1a and 2a, a water sample was also collected from the main water line entering each building for chemical and microbiological analysis at a commercial environmental testing laboratory (water analysis reports are provided in Appendix A). At least three hours after commencement of each dosing event, a fluorescent, water soluble xanthene dye, rhodamine WT (Bright Dyes FWT Red 25 Liquid, Kingscote Chemicals, Miamisburg, OH, USA), which has many applications in surface water, ground water and wastewater testing, and is certified for use in potable drinking water, was added to the stock solution (Table 1) [22]. This provided an additional opportunity, during each dosing event, to study the concentration of an additive in water available to pigs at the four drinkers over time, and to compare the passage of a highly soluble chemical product such as rhodamine WT with that of amoxicillin trihydrate, which has limited solubility [23].

All water samples collected for amoxicillin and rhodamine WT analysis were stored on wet ice until the end of each day, and then placed in a freezer at −60 °C for up to 48 h before being transported to the laboratory, allowed to thaw and then processed the same day. Each sample tube was clarified by centrifugation at 3500× *g* for 10 min and the supernatant was decanted. Triplicate 200 µL sub-samples were prepared in Thermo Scientific 96-well microplates and scanned at 274 nm for amoxicillin using a CLARIOStar Plus microplate spectrophotometer (BMG LABTECH) in UV/vis mode. Triplicate 200 µL sub-samples were also prepared in Thermo Scientific Flat Bottom 96-well black polystyrene microplates and scanned at 550/588 nm for Rhodamine WT in the CLARIOStar Plus microplate spectrophotometer in fluorescence intensity mode. Concentrations of amoxicillin and rhodamine WT in water samples were estimated using within-plate standard curves. The arithmetic mean of the triplicates for each sample was used. For amoxicillin, we plotted standard curves between 0 and 1.0 g/L and found a linear relationship (r^2^ > 0.99). The limit of detection (LOD) for amoxicillin was 0.03 g/L. For rhodamine WT, we plotted standard curves between 0 and 1.6 µL/L and found a linear relationship (r^2^ > 0.99). The LOD for rhodamine WT was 0.032 µL/L. Water samples collected for chemical and microbiological analysis were stored on ice until the end of the day, then transported by road overnight, in an icebox, to the commercial environmental testing laboratory.

The water source for buildings A and B was a blend of underground (bore) water and surface water in equal parts. Water delivered to each building during the studies was well within acceptable standards for all parameters except for iron and manganese (Building A) and iron and *Escherichia coli* (Building B). Differences in the chemical and microbiological qualities of water used were too small to be of practical relevance.

### 2.2. Simulation and Analysis of Hydraulic Behaviour of WDSs during In-Water Dosing Events

To simulate and analyse the hydraulic behaviour of the WDS in each building in each study we used EPANET, a public domain, Windows-based WDS modelling package, developed and maintained by the United States Environmental Protection Agency, that also serves as the hydraulic and water quality “engine” for many commercial WDS models used worldwide [24]. EPANET assumes that water is incompressible, pipes are closed and full, water flow is turbulent, and water flow rate and velocity are changing in each pipe section according to a water usage (demand) pattern assigned to each outlet node.

**Figure 4:**
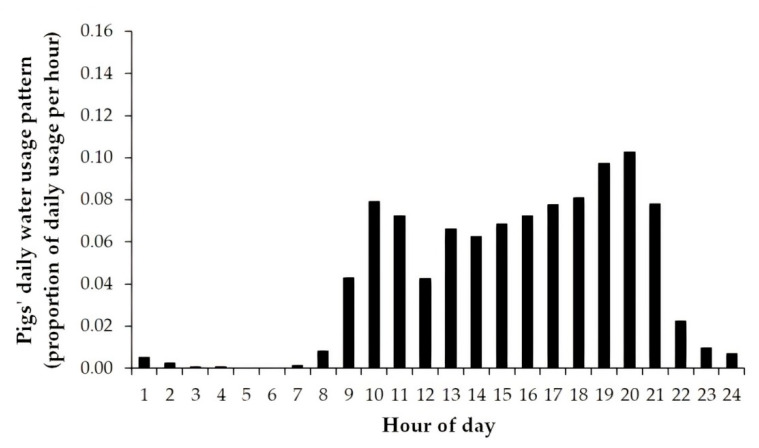
Pigs’ water usage pattern used in hydraulic WDS simulations of Studies 1a, 1b, 2a and 2b.

Network maps of the WDSs were set up in EPANET by creating scaled drawings in AutoCAD [25], a commercial computer-aided design and drafting application, and importing them into EPANET using the transitional program EPACAD [25,26]. The properties of components of the WDSs in Buildings A and B for the four studies were then specified and the hydraulic parameters were applied. The Darcy–Weisbach equation [27] was selected in EPANET to calculate pressure losses due to friction. Other inputs were the base demand (number of pigs) using each drinker, the pigs’ daily water usage pattern described in hourly increments and the head/flow curve appropriate for the specifications of the circulator pump used in Study 2b. A value of 90 mL/kg pig bodyweight was assumed for daily water consumption, as this is within the range of values found in recent studies [1,28]. Observed hourly water usage data, measured by an ultrasonic flow meter in Building B on a day three weeks prior to studies 2a and 2b, were supplied to the simulations (Figure 4). Hydraulic settings and properties of pipes, nodes, reservoirs and pumps used in the EPANET simulations are available in the Supplementary Materials.

Extended period simulations (72 h duration) were then conducted in EPANET, generating model predictions on the water age (hours) over the 72 h period, in 5-min increments, at each of four selected drinking nodes in each WDS, corresponding to L2, L3, L4 and L5 in the relevant study. These predictions were then exported from EPANET into Excel and used to generate predictions of amoxicillin and rhodamine WT concentrations at each drinker over each dosing event studied, based on its commencement time and duration. The data visualization package ggplot2 was used in R to generate amoxicillin and rhodamine WT concentration–time plots at each drinker, over each dosing event [29,30]. For each study, the observed data (based on analysis of water samples) and predicted data (based on EPANET modelling) were plotted on the same chart for ease of comparison.

## 3. Results

### 3.1. Studies 1a and 1b in Building A

Samples from four drinkers (L2, L3, L4 and L5) in Studies 1a and 1b were analysed for the concentration of amoxicillin and rhodamine WT over time. The concentrations of the solutes in the water samples were characterised by distinct phases: an initial lag after dosing commenced, followed by a rapid ascent, a period over which the concentration was relatively steady and then a rapid descent. The ascent and descent phases at each drinker were completed in ≤20 min, consistent with the plug flow reactor model described in fluid mechanics, which assumes that a medium (such as an antimicrobial) introduced to a pipe flows through the pipe as a series of infinitely thin coherent “plugs” [31]. The medium is uniformly mixed in any cross-section (plug) at any point along the pipe, but no mixing of the medium occurs in the axial direction.

**Figure 5:**
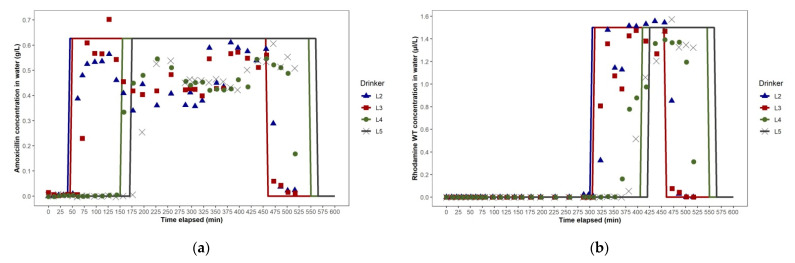
Study 1a dosing event in Building A: (**a**) amoxicillin concentration—time plots for drinkers L2, L3, L4 and L5, as predicted using EPANET (lines) and as observed (points), (**b**) rhodamine WT concentration—time plots for drinkers L2, L3, L4 and L5, as predicted using EPANET (lines) and as observed (points).

**Figure 6:**
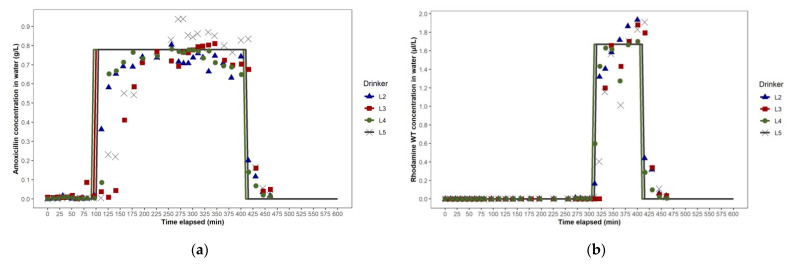
Study 1b dosing event in Building A: (**a**) amoxicillin concentration—time plots for drinkers L2, L3, L4 and L5, as predicted using EPANET (lines) and as observed (points), (**b**) rhodamine WT concentration—time plots for drinkers L2, L3, L4 and L5, as predicted using EPANET (lines) and as observed (points).

A feature of Study 1a was that steady-state concentrations of amoxicillin and rhodamine WT close to the targets of 0.62 g/L and 1.5 µL/L, respectively, were not reached in the water at any drinker (L2, L3, L4 and L5 (Figure 5a,b). In contrast, in Study 1b, the amoxicillin and rhodamine WT concentrations were maintained in a steady-state at close to the targets of 0.78 g/L and 1.67 µL/L, respectively (Figure 6a,b). The instability in Study 1a was caused by leakage through a small fissure in the plastic tube transporting the stock solution from the dosing pump to the injection point in the water line that developed 75 min after dosing commenced. The leakage effectively reduced the volume of solution injected into the water line with each pumping cycle. After 300 min, the leaking tube was replaced by the farm manager, and amoxicillin product concentrations at the drinkers nearest to the dosing pump, L2 and L3, subsequently approached the target concentration, followed by the two drinkers further from the dosing pump, L4 and L5. The leaked stock solution was captured in the stock solution container and re-injected during the dosing event. This extended the duration of the dosing event (i.e., the time taken to inject all the stock solution into the water line) beyond the end of normal daily working hours, when farm access was restricted. Consequently, it was not possible to continue to collect samples beyond 516 min and measure the amoxicillin and rhodamine WT concentrations at drinker L5 to fully assess their descent phase.

There was good concordance between the phases of the amoxicillin concentration–time plots for water at each selected drinker in Studies 1a and 1b based on the analysis of the water samples and the predictions generated from the EPANET hydraulic model simulations (Figure 5a and Figure 6a). The overall shape of the predicted amoxicillin concentration over time was very similar to that observed, even if the timings of the ascent and descent phases were sometimes a little early or later than observed. There was also good concordance between the phases of the rhodamine WT concentration–time plots for water at each drinker, based on analyses of the water samples and predictions from the simulations (Figure 5b and Figure 6b). In Study 1a, the amoxicillin and rhodamine WT concentration–time plots for water at drinkers L2 and L3 were closely aligned. However, the amoxicillin and rhodamine ascent phases observed at L5 lagged a little behind those at L4. This minor imbalance between the left and right arms of the looped WDS was also predicted by EPANET. We hypothesised that the imbalance was due to two factors: (1) the distance from the junction where water entered the loop to the furthest junction in the loop via the right arm was 8.8 m greater than via the left arm (115.6 m versus 105.8 m), and (2) the number of pigs drawing water from the right arm of the loop was 54 fewer than the number drawing water from the left arm (702 versus 756), resulting in 7% lower water demand by pigs from the right arm than from the left arm.

**Figure 7:**
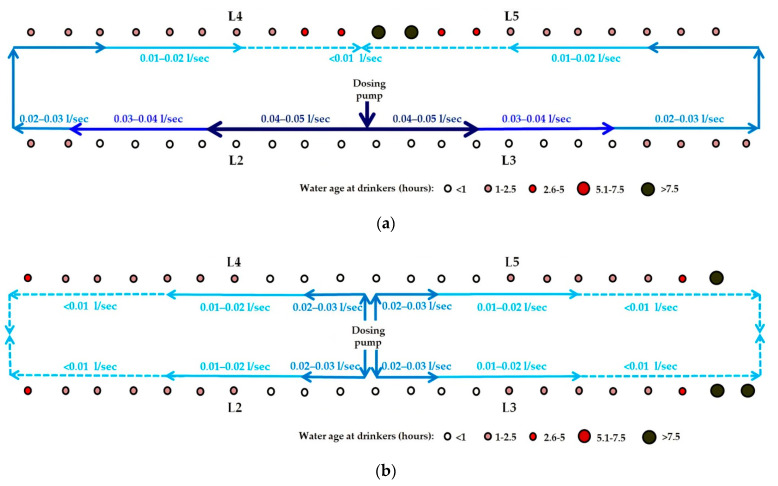
Network maps generated in EPANET, representing the WDSs in Building A at 12:00 noon (day 2) during: (**a**) Study 1a; and (**b**) Study 1b. Coloured arrows indicate the direction of water flow in each pipe section. Water flow rate (L/sec) along each pipe section is indicated by the size of the arrow and thickness of the line. The age of the water (in hours) at each drinker is indicated by the size and colour of the circle.

We did not measure volumetric flow rate and velocity at different points in the looped WDS during the studies. However, as shown in the simulations of the WDSs used for dosing in Studies 1a and 1b, we expect that, at any given time of day, as water travels further along each arm of the looped WDS from the junction at which it entered, its volumetric flow rate (L/sec) and velocity (m/sec) progressively decrease (Figure 7a,b). This, theoretically, would be a consequence of the smaller number of pigs drawing water downstream of each pipe section. Frictional losses in pressure may also have contributed. In Studies 1a and 1b, in the pipe sections furthest from the water entry point in each loop, where water travelling along each arm of the loop converged, we expect that the volumetric flow rate and velocity are close to zero, as shown in the simulations. In the dual loop WDS used in Study 1b, the distance that water needed to travel from the entry point to the furthest point in each loop was half of that for the large single-loop WDS used in Study 1a. However, in the dual loop WDS, pigs’ water demand in each loop was also halved. At any time of day, we expect that water, therefore, travels through pipe sections along each arm of each of the dual loops at approximately half the velocity that it travels along each arm of the single loop system, as shown by the network maps generated in EPANET (Figure 7a,b). This is evidenced by the longer initial lags after dosing commenced and before amoxicillin first reached drinkers L2 and L3 in Study 1b (Figure 6a), as compared to those seen in Study 1a (Figure 5a). Comparison of the EPANET simulations for Studies 1a and 1b showed that in each WDS the range of ages of water at each drinker over time were very similar, and that configuring the WDS in Building A as dual loops, rather than as one large, single loop, did not offer any advantage during antimicrobial dosing events.

### 3.2. Studies 2a and 2b in Building B

**Figure 8:**
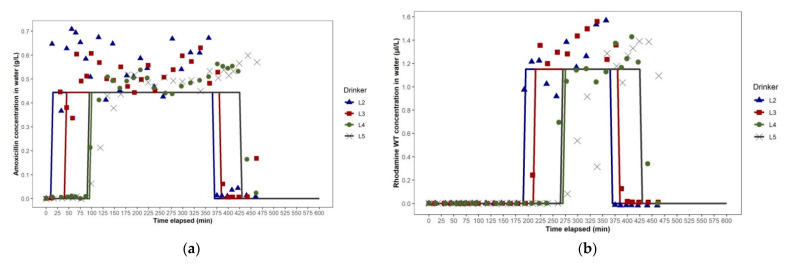
Study 2a dosing event in Building B: (**a**) amoxicillin concentration—time plots for drinkers L2, L3, L4 and L5, as predicted using EPANET (lines) and as observed (points), (**b**) rhodamine WT concentration—time plots for drinkers L2, L3, L4 and L5, as predicted using EPANET (lines) and as observed (points).

In Study 2a, the dosing event conducted in Building B with the single-looped WDS with the water entry point at the south-west corner, the amoxicillin and rhodamine WT concentration–time plots were not as expected. Even though drinker L5 was closer to the dosing pump than L4 (Figure 2c), amoxicillin and rhodamine WT first reached L5 after reaching L4, and, following the end of the dosing event, L5 passed from the steady-state phase to the rapid descent phase after L4 (Figure 8a,b). This indicated that the loop was unbalanced, with more water entering the loop travelling in a clockwise direction than in an anti-clockwise direction, i.e., the point at which water flowing along the two arms of the loop converged was not at the furthest point in the loop from the entry point. This hydraulic behaviour was consistent with the presence of a partial obstruction in the top arm of the loop to the near left of L5. A T-junction had been fitted in the waterline close to L5, to which a short pipe (approximately 15 cm long) with a gate valve was attached to facilitate periodic flushing of the WDS between batches of pigs. It is plausible that an accumulation of material at this T-junction may have constricted flow through this section of the loop. In the network map of the WDS in Building B set up in EPANET, a throttle control valve was installed near L5 to mimic such a partial obstruction to water flow, and this resulted in hydraulic behaviour consistent with that observed in Study 2a.

**Figure 9:**
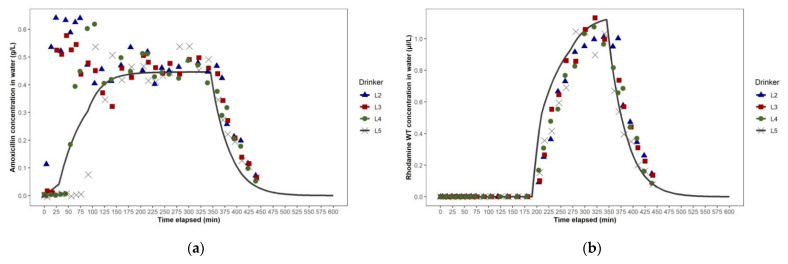
Study 2b dosing event in Building B: (**a**) amoxicillin concentration—time plots for drinkers L2, L3, L4 and L5, as predicted using EPANET (lines) and as observed (points), (**b**) rhodamine WT concentration—time plots for drinkers L2, L3, L4 and L5, as predicted using EPANET (lines) and as observed (points).

**Figure 10:**
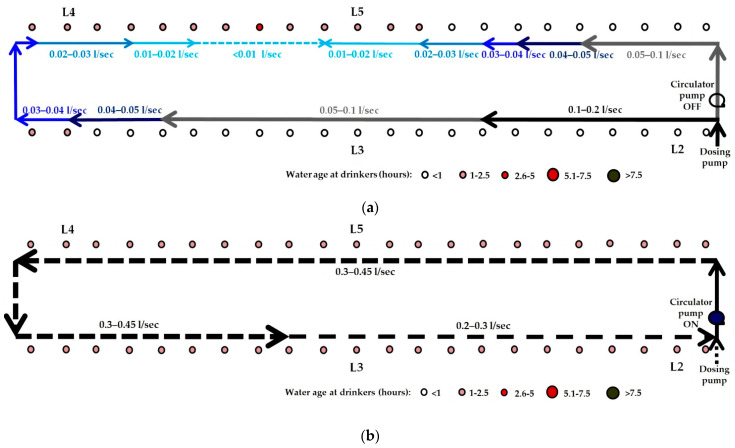
Network maps generated in EPANET, representing the WDSs in Building B at 12:00 noon (day 2) during: (**a**) Study 2a; and (**b**) Study 2b. Coloured arrows indicate the direction of water flow in each pipe section. Water flow rate (L/sec) along each pipe section is indicated by the size of the arrow and thickness of the line. The age of the water (in hours) at each drinker is indicated by the size and colour of the circle.

In Study 2b, the electric wet rotor circulator pump installed in Building B’s looped WDS was switched on to the highest of three settings approximately 10 min before the dosing event commenced. The pump appeared to have no effect on solute distribution over the first 120 min, as the ascent phases of the amoxicillin concentration–time plots at drinkers L2, L3, L4 and L5, based on analysis of water samples, were very similar in time and slope to those seen in Study 2a. However, in their steady-state phases, the amoxicillin concentration–time plots for L2, L3, L4 and L5 were much more closely aligned with each other than in Study 2a, and remained so in their descent phases (Figure 9a). The effect of the circulator pump on medicated water flow through the WDS was more clearly shown by the rhodamine WT concentration–time plots of water at drinkers L2, L3, L4 and L5, as rhodamine WT was added 195 min after the commencement of amoxicillin dosing, when the amoxicillin concentration–time plots indicated that a unidirectional flow around the building’s looped WDS had been well established. The rhodamine WT concentration–time plots for L2, L3, L4 and L5 showed a negligible initial lag and synchrony in rhodamine WT concentration at all four drinkers throughout their ascent, steady-state and descent phases (Figure 9b). This was due to the much faster flow through the WDS with the circulator pump operating. The network map generated in EPANET, representing the WDS in Building B at 12:00 noon during Study 2b, showed the anti-clockwise direction of flow. Water flow rate in the pipe section immediately downstream of the circulator pump ranged from 0.38 to 0.43 L/sec over the dosing period (Figure 10b). This equated to the circulation of the entire volume of water held in the looped WDS at any one time (approximately 450 L) around the loop over five times per hour.

For Study 2b, a different method had to be used to generate predicted amoxicillin and rhodamine WT concentration–time plots at L2, L3, L4 and L5. Given the high rate of water flow through the WDS, the concentration–time plots at L2, L3, L4 and L5 were assumed to be identical, and the law of mass conservation, which states that inflows, outflows and change in storage of mass in a system must be in balance, was applied [31,32]. The concentrations of amoxicillin or rhodamine WT at L2, L3, L4 and L5 during each successive 5 min period of the dosing event were calculated using two formulae:

Q_t_ = Q_e_ − Q_l_ + Q_c_, (1)

where Q_t_ is the total quantity of solute circulating in the looped WDS, Q_e_ is the quantity of solute entering the WDS (a function of the concentration of solute entering the WDS and pigs’ water demand), Q_l_ is the quantity of solute leaving the system (a function of concentration of solute already circulating in the WDS and pigs’ water demand), and Q_c_ is the quantity of solute circulating in the WDS at the end of the previous 5-min period.

C_t_ = Q_t_/V, (2)

where C_t_ is the concentration of solute circulating in the looped WDS, Q_t_ is the total quantity of solute circulating in looped WDS, and V is the volume of water resident in the looped WDS at any one time.

Two features of the amoxicillin and rhodamine WT concentration–time plots in Study 2b, which contrasted with previous studies, were the very short initial lag after dosing commenced, and the long ascent phase, which occurred over 150 min as the pigs’ water usage drew the amoxicillin and rhodamine WT into the loop. The slope of this ascent phase was a function of the concentrations of the stock solution of amoxicillin and rhodamine WT, the dosing pump’s injection ratio, the pigs’ water usage and the volume of water resident in the looped WDS at any one time.

In Studies 2a and 2b, marked fluctuations in the concentrations of amoxicillin and rhodamine WT occurred at each drinker throughout both dosing events, deduced by the analysis of water samples; however, these fluctuations were masked in Study 2b by the high water flow rates in the WDS. The fluctuations were associated with periods when water usage by pigs exceeded 500 L/h, as the model of the electric dosing pump used in Building B had a maximum pumped output of 4.7 L/h (compared to 36 L/h for the model of dosing pump used in Building A). During periods of high usage, the dosing pump was observed to be pumping continuously, and its screen indicated ‘High Flow’. The actual injection ratio would have been lower than the programmed ratio of 1:100 for short periods, resulting in under-dosing. However, despite the dosing pump’s limited pumping capacity, the amoxicillin concentration remained above target (0.45 mg/mL) at L2, L3, L4 and L5 for most of the dosing periods (albeit with marked fluctuations).

## 4. Discussion

There were two main findings from this study: (1) when dosing a group of pigs in a building, the concentration of antimicrobial in the water available at drinkers in each pen over time was a function of water flow rates in each pipe section of the WDS, as determined by the WDS’s characteristics and pigs’ water usage and drinking patterns; and (2) it is feasible to correct for the natural hydraulic behaviour of a looped WDS during a dosing event using a circulator pump, and thereby eliminate differences in the antimicrobial concentration at drinkers, throughout a building, over time.

### 4.1. Factors Influencing the Concentration of Antimicrobial in Water Available to Pigs at Drinkers over Time

This study has provided a fundamental understanding of the hydraulic behaviour of looped WDSs in pig buildings and has demonstrated how it influences the concentration of an antimicrobial in the water available to pigs at drinkers located throughout a pig building, both during and after an in-water dosing event. Water flow in looped WDSs is defined as turbulent, characterized by random and rapid fluctuations of swirling regions of fluid, called eddies, that create fluctuations in the flow velocity and pressure. Turbulent flow ensures that when a concentrated antimicrobial stock solution is injected into the main pipe of a pig building’s WDS, it is rapidly dispersed within the column of water as it flows through the pipe. The steep slopes of the ascent phase and descent phases of the amoxicillin and rhodamine WT concentration–time plots in Studies 1a, 1b and 2a are consistent with the velocity profile that occurs in a turbulent flow [33].

The two buildings in which we conducted our studies had looped WDSs comprising 50 mm internal diameter pipes, as is common in many weaner and grower/finisher buildings on pig farms. According to the equation Q = (π ∗ r2) × V, where Q = volumetric flow rate, r = pipe radius and V = water velocity, water drawn through a section of pipe in a building’s WDS with an internal diameter of 50 mm—at the flow rate required to meet the pigs’ water demand—travels at one quarter of the average velocity that it would through a pipe that is 25 mm in internal diameter. Our hydraulic modelling of WDSs in Buildings A and B during Studies 1a, 1b and 2a showed that in the 50 mm internal diameter pipe sections of the loop nearest to the dosing pump, water velocities over each 24 h period never exceeded 0.1 m/sec; this was even during periods of peak water demand by pigs. Furthermore, velocities were considerably lower further along each arm of the loop. These water velocities are lower than those found in most municipal WDSs used to deliver potable water to residential and industrial premises. When designing municipal WDSs, a minimum velocity of at least 0.5 m/sec is generally specified, to ensure that water does not reside in the system for too long, thus preventing deterioration in water quality and accumulations of sediments [34,35,36]. Our studies have indicated that WDSs in pig buildings comprising 50 internal diameter mm pipes, as found on many pig farms, are likely to be ‘over-sized’ relative to their typical ‘peaking factor’, i.e., maximum daily usage rate divided by the average daily usage rate [37]. While over-sizing a building’s WDS helps to ensure satisfactory volumetric flow rates from all drinkers, including periods of high demand (provided the system’s operating pressure is adequate), it results in a WDS that has a large holding volume relative to the pigs’ daily water demand, with very low water velocities. During an in-water dosing event, this may contribute to large differences between drinkers; both in the initial lag after commencement of dosing but before an antimicrobial first reaches the drinker, and in the duration over which the antimicrobial is available at the drinker. If, for any reason, a weaner or grower/finisher building is stocked below its designed capacity, this will further reduce total water demand, and therefore water flow rates and velocities through pipe sections of its WDS. This may be the case on farms that have reduced their pig stocking densities in buildings to comply with an updated industry code of practice or a standard set by a retailer or animal welfare organisation.

We have shown that operating a circulator pump in a pig building’s looped WDS to establish and maintain high, steady, uni-directional water flow during a dosing event enables differences in the antimicrobial dose delivered to pigs at drinkers in pens throughout the building and ingested over time by pigs to be largely eliminated. The costs to install a circulator pump in each building on a farm with a looped WDS and run it during dosing events may be justified in situations where dosing would result in large differences in the hourly rates of antimicrobial ingestion of pigs at different points along the WDS when using a conventional demand-driven approach. The high water flow generated using the circulator pump may also help to disperse any sediment that may have accumulated at points within the WDS. There are several factors associated with the use of a circulator pump in a looped WDS that farm managers should consider before opting for this approach. The pump must be installed correctly and connected to a power source. The farm manager must remember to switch the pump on before a dosing event is commenced and switch it off when dosing is finished. The pump will ultimately wear out and need to be replaced. It is preferable to use a circulator pump with a control panel that displays the current water flow rate based on pump load, so that this can be monitored. The gradual increase in antimicrobial concentration over >2 h, that occurs throughout a looped WDS when using a circulator pump during dosing, is undesirable; the resultant low hourly rates of antimicrobial consumption by pigs over the first few hours would lead both to a slower rise in antimicrobial concentration at the site of infection to levels above the minimum inhibitory concentration (MIC), and to a later attainment of the PK/PD target for the antimicrobial that best predicts its efficacy. However, farm managers and veterinarians could readily address this issue by incorporating a loading dose into the dosing regimen and commencing daily dosing events at the beginning of a period of moderate to high pig water consumption. This would require on-farm measuring systems that provide farm managers and veterinarians with easily interpretable data on the water wastage and consumption pattern of each group of pigs being dosed.

As we have previously reported, many farm managers encounter challenges in dissolving amoxicillin trihydrate products in dosing pump stock solution containers or header tanks [18]. However, as observed in Studies 1b, 2a and 2b, target concentrations of amoxicillin of 0.78 mg/mL and 0.45 mg/mL were reached and sustained for substantial periods at each of the four drinkers sampled during dosing. From this, we can conclude that: (1) the amoxicillin trihydrate product used was well-dissolved and -dispersed in the WDSs, even though suspended particles were seen in the dosing pump stock solution early in the dosing events; (2) medicated water passed down the entire length of the WDSs without being diluted; and (3) the amoxicillin trihydrate product used remained stable in the stock solutions for the duration of the dosing events and as it passed through the WDSs to each drinker, after being injected into the waterline. As water quality analyses showed (Appendix A), the concentration of iron in the drinking water in each study was above the acceptable water quality standard for consumption by pigs of <0.3 mg/L [38]. Amoxicillin has been shown to form complexes with metals, including iron. However, during our studies, we did not detect any reduction in the concentration of amoxicillin in water collected at drinkers over the duration of each dosing event (ranging from 4:47 h to 7:22 h). This is consistent with a laboratory study reported by Edwards (2018) [38] which found that another soluble antimicrobial product containing amoxicillin (870 g/kg active) as the trihydrate (Sol-U-Mox Amoxycillin Soluble Powder, Bayer Australia Ltd., Pymble, NSW, Australia) remained highly stable after mixing for 24 h in water with an iron concentration of 0.73 mg/L.

This study has demonstrated the importance of ensuring that, when administering an antimicrobial product with low solubility, such as amoxicillin trihydrate, to a group of pigs using a proportional dosing pump, the dosing pump type and model is suitable for the WDS’s operating pressure, and that, if the pump is electric-powered, its pumped output (L/h) can cope with pigs’ water usage during the dosing period, given their number and bodyweight. It is unfortunate that farm managers and veterinarians do not have access to detailed protocols for preparing and using stock solutions of specific antimicrobial products in dosing pumps, either from manufacturers of antimicrobial products or those of dosing pumps. Such protocols should be provided on product labels and brochures. Preparation of an excessively concentrated stock solution of an antimicrobial with low solubility, such as amoxicillin as the trihydrate, should be avoided by mixing the quantity of antimicrobial product to be administered in a sufficient volume of water. Sodium carbonate should be added to an amoxicillin trihydrate stock solution to elevate its pH to >8, at which the solubilisation of amoxicillin trihydrate is greatly enhanced [39]. The stock solution should be continuously agitated during dosing using a small submersible pump, magnetic stirrer or other device to ensure that any undissolved drug is well-dispersed in the stock solution.

### 4.2. Further Concerns Associated with Over-Sized Pig Building WDSs

Low velocity water flows that occur in pipe sections of over-sized pig building WDSs also influence biofilm growth [40]. Studies have shown that, when compared to WDSs with high velocity water flows (>0.3 m/sec), those with low velocity flows have lower hydrodynamic shear forces at the internal surface of their pipes, which leads to growth of thicker, less dense and less stable biofilms [41,42,43]. Further to this, low velocity water flows through pipes lead to higher concentrations of antimicrobial-resistant bacteria (ARB) and a higher prevalence of antimicrobial-resistant genes (ARGs), and different microbial community compositions than are found in pipes with higher velocity water flow [44,45,46]. Exposure of microbial communities in biofilms to sub-inhibitory antimicrobial concentrations, which is likely in pig building WDSs, especially during lower metaphylactic in-water dosing events conducted over >12 h, could also modulate biofilm matrix composition. This would lead to greater biofilm persistence and greater protection within the biofilm against antimicrobials, accelerating the bacterial conjugation and spread of ARGs throughout a wider range of bacterial species [47,48,49].

Water temperatures have been found to be higher in larger-diameter pipes in pig building WDSs, due to lower water velocities which provide more time for the water to absorb heat from the environment [50]. Farm managers’ concerns about high water temperatures in pipes on hot summer days are well-founded. In a controlled experiment, the average daily gain of 10-week-old pigs offered water at 28.4 °C was 17% less than pigs offered water at 17.8 °C [50]. Higher water temperatures in pipes have also been positively correlated with biofilm growth in WDSs [40].

### 4.3. Limitations of the Study

During these prospective observational studies of in-water dosing events, we were not able to measure water flow rate at any point in the building’s WDS, and, because of the time required to collect frequent water samples, we were limited to sampling four drinkers within each building. While we found good concordance between the amoxicillin and rhodamine WT concentration–time plots of water at each selected drinker in the studies based on analysis of water samples and those generated from the EPANET hydraulic model simulations, use of real-time water flow data measured in each building at the time of the studies in simulations may improve concordance. We are also conscious that, while pigs drink in short bouts, hydraulic WDS simulation models are unable to simulate short, randomly distributed water usage events within each one-hour period. This study should be considered to be a first step in gaining a detailed understanding of the hydraulic behaviour of looped pig building WDSs and the factors that influence the concentration of an antimicrobial in the water available to pigs at drinkers throughout a pig building, during and after an in-water dosing event.

## 5. Conclusions

The spatial and temporal variations in water age (residence time) at drinkers in a pig building can be calculated with reasonable certainty by representing the building’s WDS in a hydraulic model, specifying all necessary model inputs and running extended period simulations. These data can then be used to evaluate in-water dosing regimens. We have shown how the concentration of an antimicrobial in the water available to pigs at drinkers in each pen over time, during and after a dosing event (the first source of between-animal variation in systemic exposure to an antimicrobial), can be estimated using hydraulic WDS modelling. This provides an opportunity to calculate the predicted consumption of medicated water by pigs in each pen over time (the second source of between-animal variation in systemic exposure to the antimicrobial), and then use these predictions as a dosing schedule in a population pharmacokinetic (PPK) model for the antimicrobial, in order to compare the systemic exposures to the antimicrobial achieved by pigs using drinkers throughout the building. Alternative dosing regimens, based on different WDS characteristics, water consumption patterns and dosing practices, could be evaluated using this three-step process, and an optimal dosing regimen could be determined. Our findings are also applicable to other additives that are administered to pigs in drinking water offered ad libitum, for which the degree of efficacy is dependent on the dose administered. These additives include vaccines, parasiticides, organic acids, electrolytes, minerals, vitamins, amino acids, sweeteners, direct-fed microbials, essential oils and potential new therapeutic products, such as bacteriophages.

Given that the WDS in a pig building is driven by the water usage of pigs throughout the building over time, it is important that temporal variations in water usage over the period being modelled are as realistic as possible. More research is needed to characterise weaner and grower/finisher pigs’ diurnal patterns of water usage (consumption and wastage) and understand the extent to which they vary from day-to-day within the same group of pigs and between groups of pigs under different conditions. If these patterns are found to vary substantially within and between groups of pigs over a daily timescale, and therefore cannot be reliably predicted, then determining the optimal dosing regimen would require farm managers and veterinarians to have access to on-farm systems that measure the daily water usage of each group of pigs to be dosed. While farm managers have little or no control over the daily water usage pattern of a group of pigs, they are able to modify the characteristics of the WDS in the pigs’ building and their own in-water dosing practices. The dosing commencement time and duration selected by the farm manager and veterinarian, the water usage pattern of the group of pigs over the dosing event and characteristics of the building’s WDS will determine how different the hourly rates of antimicrobial ingestion of pigs are throughout the building, and, ultimately, what proportion of pigs in the building attain the systemic exposure to the antimicrobial that is required for high clinical efficacy and the suppression of emergent antimicrobial resistance.

## Figures and Tables

**Table 1:** Descriptions of Studies 1a, 1b, 2a and 2b.

	Studies 1a and 1b	Studies 2a and 2b
Building	‘A’ Concrete floor Natural ventilation 43 wet/dry feeders	‘B’ Concrete floor Natural ventilation 44 wet/dry feeders
Pig flow	All-in, all-out Entry: 25 kg Exit: 95 kg	All-in, all-out Entry: 25 kg Exit: 95 kg
WDS in building	Large single loop with central water entry point (1a) Main pipeline: PVC 50 mm internal diameter (less than one year old) Gate valves installed in loop to convert WDS into smaller dual loops (1b)	Large single loop with offset water entry point (2a and 2b) Main pipeline: PVC 50 mm internal diameter (less than one year old) Circulator pump operating in loop (2b) (DAB A 80/180 XM ^^^)
Antimicrobial product administered	Amoxicillin (870 g/kg) as the trihydrate ^#^	Amoxicillin (870 g/kg) as the trihydrate ^#^
Pigs dosed	1460 male and female pigs Average bodyweight: 41 kg	2200 male and female pigs Average bodyweight: 53 kg
Dose of active antimicrobial administered	26 mg/kg bodyweight	10 mg/kg bodyweight
Dosing events conducted (on consecutive days)	Study 1a (Large single loop): Start, 7:39 a.m.; duration, 7:22 h Study 1b (Smaller dual loops): Start, 7:20 a.m.; duration, 6:04 h	Study 2a (circulator pump off): Start, 7:50 a.m.; duration, 6:05 h Study 2b (Circulator pump on): Start, 7:27 a.m.; duration, 5:47 h
Dosing equipment used	Electric dosing pump (Select 640 *) Plastic stock solution container (70 L capacity) Small submersible pump for agitation	Electric dosing pump (Select 380 *) Plastic stock solution container (70 L capacity) Small submersible pump for agitation
Stock solution volume	Study 1a: 25 L Study 1b: 20 L	Study 2a: 26 L Study 2b: 26 L
Antimicrobial product concentration in stock solution	Study 1a: 62 g/L Study 1b: 78 g/L	Study 2a: 45 g/L Study 2b: 45 g/L
Rhodamine WT ^+^ concentration in stock solution	Study 1a: 115 µL/L (added at 12:22 p.m.) Study 1b: 167 µL/L (added at 11:41 a.m.)	Study 2a: 115 µL/L (added at 10:55 a.m.) Study 2b: 115 µL/L (added at 10:41 a.m.)
Dosing pump injection rate	1:100 (*v*/*v*)	1:100 (*v*/*v*)
Target antimicrobial product concentration in building’s WDS	Study 1a: 0.62 g/L Study 1b: 0.78 g/L	Study 2a: 0.45 g/L Study 2b: 0.45 g/L
Target rhodamine WT ^+^ concentration in building’s WDS	Study 1a: 1.50 µL/L Study 1b: 1.67 µL/L	Study 2a: 1.15 µL/L Study 2b: 1.15 µL/L
Drinkers sampled and studied, and their distance from dosing pump in direction of flow around loop(s) as indicated in Figure 2a–c	Study 1a: L2 (node 20): 28.5 m L3 (node 36): 29.5 m L4 (node 60): 98.4 m L5 (node 76): 107.2 m Study 1b: L2 (node 20): 28.5 m L3 (node 36): 29.5 m L4 (node 60): 28.5 m L5 (node 76): 29.5 m	Study 2a: L2 (node 46): 9.1 m L3 (node 36): 57.0 m L4 (node 50): 122.3 m L5 (node 68): 67.6 m Study 2b: L2 (node 46): 220.5 m L3 (node 36): 174.4 m L4 (node 50): 109.1 m L5 (node 68): 67.6 m

^#^ AbbeyMox Amoxicillin Soluble Powder, Abbey Animal Health Pty. Ltd., Glendenning, NSW, Australia. ^+^ Bright Dyes FWT Red 25 Liquid, Kingscote Chemicals, Miamisburg, Ohio, USA. * Dosing Solutions Ltd., Clavering, Saffron Walden, UK. ^^^ DAB Pumps, Mestrino, Padova, Italy.

## Data Availability

The data presented in this study are available on request from the corresponding author. The data are not publicly available because of the conditions of the ethics agreement.

## Supplementary Materials

The following are available online at https://www.mdpi.com/article/10.3390/ani11082362/s1, Table S1: Default hydraulic settings and properties of pipes, nodes, reservoirs and pumps used in EPANET simulations.

## Appendix A

**Table A1:** Water quality analyses—Study 1a in Building A *^.

Test	Observation	Unit	LQL ^#^
Chloride	3.9	mg/L	1
pH (at 25 °C)	6.9	pH units	0.1
Sulphate (as SO_4_)	<5	mg/L	5
Total Dissolved Solids (Dried at 180 °C ± 2 °C)	42	mg/L	10
Hardness mg equivalent CaCO_3_/L	16	mg/L	5
Alkalinity (speciated):			
Bicarbonate alkalinity (as CaCO_3_)	44	mg/L	20
Carbonate alkalinity (as CaCO_3_)	<10	mg/L	10
Hydroxide alkalinity (as CaCO_3_)	<20	mg/L	20
Total alkalinity (as CaCO_3_)	44	mg/L	20
Heavy Metals:			
Arsenic	0.003	mg/L	0.001
Cadmium	<0.0002	mg/L	0.0002
Chromium	0.001	mg/L	0.001
Copper	0.023	mg/L	0.001
Iron	1.7	mg/L	0.05
Lead	<0.001	mg/L	0.001
Manganese	0.13	mg/L	0.005
Mercury	<0.0001	mg/L	0.0001
Molybdenum	<0.005	mg/L	0.005
Nickel	<0.001	mg/L	0.001
Zinc	<0.005	mg/L	0.005
Alkali Metals:			
Calcium	3.3	mg/L	0.5
Magnesium	1.7	mg/L	0.5
Potassium	1.2	mg/L	0.5
Sodium	5.1	mg/L	0.5
Pathogens:			
*Escherichia coli*	9.0	MPN/100 mL	1
Total coliforms	260	MPN/100 mL	1

Comments: Iron concentration was well above the acceptable standard for pigs (<0.3 mg/L) [50]. Manganese concentration was above the acceptable standard for pigs (<0.1 mg/L) [50]. Water was well within acceptable standards for all other parameters tested [50]. * Full analysis performed at NATA-accredited environmental laboratory (Eurofins Environmental Laboratory, 6 Monterey Road, Dandenong South, Victoria 3175, Australia). ^ Water samples were collected from tap in main water line near dosing pump. ^#^ Lower quantification limit, which is the smallest value returned by the laboratory.

**Table A2:** Water quality analyses—Study 2a in Building B *^.

Test	Observation	Unit	LQL ^#^
Chloride	42	mg/L	1
pH (at 25 °C)	7.9	pH units	0.1
Sulphate (as SO_4_)	<5	mg/L	5
Total Dissolved Solids (Dried at 180 °C ± 2 °C)	36	mg/L	10
Hardness mg equivalent CaCO_3_/L	13	mg/L	5
Alkalinity (speciated):			
Bicarbonate alkalinity (as CaCO_3_)	39	mg/L	20
Carbonate alkalinity (as CaCO_3_)	<10	mg/L	10
Hydroxide alkalinity (as CaCO_3_)	<20	mg/L	20
Total alkalinity (as CaCO_3_)	39	mg/L	20
Heavy Metals:			
Arsenic	0.002	mg/L	0.001
Cadmium	<0.0002	mg/L	0.0002
Chromium	0.001	mg/L	0.001
Copper	0.006	mg/L	0.001
Iron	0.77	mg/L	0.05
Lead	<0.001	mg/L	0.001
Manganese	0.041	mg/L	0.005
Mercury	<0.0001	mg/L	0.0001
Molybdenum	<0.005	mg/L	0.005
Nickel	<0.001	mg/L	0.001
Zinc	0.006	mg/L	0.005
Alkali Metals:			
Calcium	2.7	mg/L	0.5
Magnesium	1.5	mg/L	0.5
Potassium	1.0	mg/L	0.5
Sodium	3.7	mg/L	0.5
Pathogens:			
*Escherichia coli*	330	MPN/100 mL	1
Total coliforms	>2400	MPN/100 mL	1

Comments: Iron concentration was above the acceptable standard for pigs (<0.3) [50]. *E. coli* level was well above the acceptable standard for pigs (<50 MPN/100 mL) [50]. Water was well within acceptable standards for all other parameters tested [50]. * Full analysis performed at NATA-accredited environmental laboratory (Eurofins Environmental Laboratory, 6 Monterey Road, Dandenong South, Victoria 3175, Australia). ^ Water samples were collected from tap in main water line near dosing pump. ^#^ Lower quantification limit, which is the smallest value returned by the laboratory.
