# Supplementary material for: Effect of Drinking Water Distribution System Design on Antimicrobial Delivery to Pigs
Source: Animals (Basel). 2021 Aug 10;11(8):2362. doi: 10.3390/ani11082362 (PMC8388689; doi:10.3390/ani11082362)
Supplement: Supplementary file 1 [file animals-11-02362-s001.zip › animals-1291887-supplementary.pdf]

Effect of drinking water distribution system design on antimicrobial delivery to pigs

Stephen Little, Andrew Woodward, Glenn Browning and Helen Billman-Jacobe

Table S1. Hydraulic settings and properties of pipes, nodes, reservoirs and pumps used in EPANET simulations.

Setting:	Value:
Default hydraulic settings:	
Flow units	L/sec
Headloss formula	Darcy-Weisbach
Specific gravity	1
Relative viscosity	1
Maximum trials	40
Accuracy	0.001
Demand multiplier - Studies 1a and 1b	0.0000427
Demand multiplier - Studies 2a and 2b	0.0000552
Main pipes:	
Diameter	50mm
Roughness	0.1
Loss coefficient	0
Drinker nodes:	
Elevation	0
Base demand – Studies 1a and 1b	36
Base demand – Studies 2a and 2b	50
Reservoir:	
Total head	15
Circulator pump:	
Flow:	Head:
0	8.3
0.5	7.6
1	6.6
1.5	5.2
2	3.65
2.6	1.6
Throttle control valve:	
Diameter	50mm
Type	TCV
Setting	4000
Loss coefficient	0.5
